# Supplementary material for: Comparative analysis of the metabolically active microbial communities in the rumen of dromedary camels under different feeding systems using total rRNA sequencing
Source: PeerJ. 2020 Oct 29;8:e10184. doi: 10.7717/peerj.10184 (PMC7603790; doi:10.7717/peerj.10184)
Supplement: Supplemental Information 1 [file peerj-08-10184-s001.docx]

**Supplementary table S1: The chemical composition (%) of diets fed to camels under investigation**

| **Feeds** | **DM** | **Chemical analysis (%) on DM basis** | | | | | | | |
| --- | --- | --- | --- | --- | --- | --- | --- | --- | --- |
|  |  | **Ash** | **CP** | **CF** | **EE** | **NFE** | **NDF** | **ADF** | **ADL** |
| **Concentrate mixture^1^** | 92.41 | 9.96 | 13.81 | 14.48 | 2.92 | 58.83 | 35.18 | 20.10 | 7.49 |
| **Fresh Egyptian Clover^2^** | 18.00 | 12.94 | 14.23 | 31.38 | 1.34 | 40.11 | 49.60 | 29.10 | 5.20 |
| **Egyptian Clover hay** | 89.29 | 12.13 | 12.44 | 25.64 | 1.73 | 48.06 | 44.15 | 38.37 | 10.50 |
| **Wheat straw** | 93.12 | 10.80 | 2.94 | 39.71 | 0.45 | 46.10 | 77.30 | 50.04 | 9.00 |

**^1^Concentrate mixture composed of 30% wheat bran, 22% cotton seed meal, 33%yellow corn, 10% sunflower meal, 3% molasses, 1.5% limestone, 0.5% salt .^2^*Trifolium alexandrinum*;**
