# Supplementary material for: Comparative analysis of the metabolically active microbial communities in the rumen of dromedary camels under different feeding systems using total rRNA sequencing
Source: PeerJ. 2020 Oct 29;8:e10184. doi: 10.7717/peerj.10184 (PMC7603790; doi:10.7717/peerj.10184)
Supplement: Supplemental Information 2 [file peerj-08-10184-s002.docx]

**Supplementary Table S2**: Metadata information of rumen solid and liquid samples of camel under different feeding systems.

| Sample | Fraction | Group | Feeding System | | | | | | | |
| --- | --- | --- | --- | --- | --- | --- | --- | --- | --- | --- |
|  |  |  | Diet | DM  Intake | CP  Intake | CF  Intake | Location | Latitude | Longitude | Climate |
| G1-1S | Solid | G1 | Concentrate  Mixture  +  Egyptian clover hay+  Wheat straw | 6.17 | 0.67 | 1.52 | Maryout Research Station,  Alexanderia, Egypt | 31.005102 | 29.789865 | warm and  temperate |
| G1-1L | Liquid |  |  |  |  |  |  |  |  |  |
| G1-2S | Solid |  |  |  |  |  |  |  |  |  |
| G1-2L | Liquid |  |  |  |  |  |  |  |  |  |
| G1-3S | Solid |  |  |  |  |  |  |  |  |  |
| G1-3L | Liquid |  |  |  |  |  |  |  |  |  |
| G2-4S | Solid | G2 | Fresh  Egyptian clover (100% high quality diet) | 6.83 | 0.97 | 2.14 | Komhammada slaughtering house,  Elbehera, Egypt | 30,767297 | 30.675392 | warm and  temperate |
| G2-4L | Liquid |  |  |  |  |  |  |  |  |  |
| G2-5S | Solid |  |  |  |  |  |  |  |  |  |
| G2-5L | Liquid |  |  |  |  |  |  |  |  |  |
| G2-6S | Solid |  |  |  |  |  |  |  |  |  |
| G2-6L | Liquid |  |  |  |  |  |  |  |  |  |
| G2-7S | Solid |  |  |  |  |  |  |  |  |  |
| G2-7L | Liquid |  |  |  |  |  |  |  |  |  |
| G2-8S | Solid |  |  |  |  |  |  |  |  |  |
| G2-8L | Liquid |  |  |  |  |  |  |  |  |  |
| G2-9S | Solid |  |  |  |  |  |  |  |  |  |
| G2-9L | Liquid |  |  |  |  |  |  |  |  |  |
| G3-10S | Solid | G3 | Wheat  Straw (100 % low quality diet) | 6.97 | 0.2 | 2.77 | Pasaten slaughtering house, Cairo,Egypt | 30.0017914 | 31.2713185 | warm and  temperate |
| G3-10L | Liquid |  |  |  |  |  |  |  |  |  |
| G3-11S | Solid |  |  |  |  |  |  |  |  |  |
| G3-11L | Liquid |  |  |  |  |  |  |  |  |  |
