# Supplementary material for: Comparative analysis of the metabolically active microbial communities in the rumen of dromedary camels under different feeding systems using total rRNA sequencing
Source: PeerJ. 2020 Oct 29;8:e10184. doi: 10.7717/peerj.10184 (PMC7603790; doi:10.7717/peerj.10184)
Supplement: Supplemental Information 3 [file peerj-08-10184-s003.docx]

**Supplementary table S3:** The relative abundance (%) of active bacterial genera in ruminal solid (SF) and liquid (LF) fractions of camels fed on

mixed ration (G1), high quality forage (G2), low quality forage (G3).

|  | Phylum/Family/ Genus | Animal | Animal | Animal | Animal | Animal | Animal | Animal | Animal | Animal | Animal | Animal | Animal | Animal | Animal | Animal | Animal | Animal | Animal | Animal | Animal | Animal | Animal |
| --- | --- | --- | --- | --- | --- | --- | --- | --- | --- | --- | --- | --- | --- | --- | --- | --- | --- | --- | --- | --- | --- | --- | --- |
|  |  | G1-1S | G1-1L | G1-2S | G1-2L | G1-3S | G1-3L | G2-4S | G2-4L | G2-5S | G2-5L | G2-6S | G2-6L | G2-7S | G2-7L | G2-8S | G2-8L | G2-9S | G2-9L | G3-10S | G3-10L | G3-11S | G3-11L |
| Phylum | Actinobacteria |  |  |  |  |  |  |  |  |  |  |  |  |  |  |  |  |  |  |  |  |  |  |
| Family | Coriobacteriaceae |  |  |  |  |  |  |  |  |  |  |  |  |  |  |  |  |  |  |  |  |  |  |
| Genus | Atopobium | 0.750 | 0.500 | 0.455 | 0.325 | 0.535 | 0.460 | 1.170 | 0.630 | 1.945 | 1.575 | 1.795 | 4.460 | 1.595 | 1.780 | 2.230 | 3.150 | 1.650 | 4.060 | 0.610 | 0.185 | 0.220 | 0.220 |
| Genus | Olsenella | 0.130 | 0.000 | 0.160 | 0.105 | 0.205 | 0.000 | 0.445 | 0.255 | 1.000 | 0.985 | 0.805 | 2.485 | 0.965 | 1.055 | 1.065 | 1.535 | 0.895 | 1.605 | 0.410 | 0.140 | 0.000 | 0.000 |
| Genus | uncultured- | 0.300 | 0.155 | 0.285 | 0.145 | 0.235 | 0.165 | 0.350 | 0.105 | 0.580 | 0.590 | 0.500 | 0.480 | 0.400 | 0.215 | 0.630 | 0.515 | 0.425 | 0.245 | 0.100 | 0.000 | 0.000 | 0.000 |
| Phylum | Phylum Bacteroidetes |  |  |  |  |  |  |  |  |  |  |  |  |  |  |  |  |  |  |  |  |  |  |
| Family | Marinilabiaceae |  |  |  |  |  |  |  |  |  |  |  |  |  |  |  |  |  |  |  |  |  |  |
| Genus | uncultured | 0.000 | 0.000 | 0.000 | 0.000 | 0.000 | 0.000 | 0.000 | 0.000 | 0.100 | 0.000 | 0.000 | 0.000 | 0.000 | 0.000 | 0.110 | 0.100 | 0.000 | 0.000 | 0.720 | 0.255 | 0.000 | 0.000 |
| Family | Prevotellaceae |  |  |  |  |  |  |  |  |  |  |  |  |  |  |  |  |  |  |  |  |  |  |
| Genus | Paraprevotella- | 0.195 | 0.245 | 0.100 | 0.190 | 0.140 | 0.280 | 0.105 | 0.475 | 0.000 | 0.000 | 0.180 | 0.410 | 0.145 | 0.560 | 0.205 | 0.675 | 0.135 | 0.545 | 0.350 | 0.265 | 0.000 | 0.000 |
| Genus | Prevotella | 3.870 | 10.050 | 6.395 | 15.370 | 6.140 | 12.110 | 2.420 | 7.490 | 3.480 | 3.550 | 3.515 | 9.565 | 3.625 | 8.360 | 2.450 | 8.675 | 3.290 | 7.880 | 7.950 | 6.175 | 2.575 | 2.575 |
| Genus | Xylanibacter | 0.850 | 1.200 | 0.795 | 1.510 | 0.570 | 1.155 | 0.370 | 1.055 | 0.440 | 0.375 | 0.570 | 1.020 | 0.575 | 1.555 | 0.510 | 1.185 | 0.500 | 1.095 | 1.475 | 3.625 | 2.125 | 2.125 |
| Genus | uncultured | 7.015 | 5.530 | 2.870 | 3.830 | 2.970 | 3.150 | 2.820 | 3.095 | 2.525 | 2.280 | 5.060 | 3.165 | 3.105 | 3.200 | 2.880 | 4.200 | 1.780 | 4.570 | 5.610 | 3.300 | 2.510 | 2.510 |
| Family | Rikenellaceae |  |  |  |  |  |  |  |  |  |  |  |  |  |  |  |  |  |  |  |  |  |  |
| Genus | RC9_gut_group | 2.100 | 2.985 | 1.620 | 1.890 | 1.980 | 1.880 | 1.940 | 1.275 | 1.235 | 1.305 | 0.745 | 1.415 | 0.895 | 1.255 | 1.940 | 1.655 | 1.400 | 1.525 | 2.625 | 6.135 | 2.730 | 2.730 |
| Genus | uncultured | 0.000 | 0.000 | 0.115 | 0.145 | 0.150 | 0.100 | 0.260 | 0.185 | 0.130 | 0.130 | 0.000 | 0.180 | 0.000 | 0.110 | 0.150 | 0.200 | 0.115 | 0.170 | 0.690 | 7.170 | 2.675 | 2.675 |
| Genus | Cytophagia | 0.460 | 0.515 | 0.470 | 0.470 | 0.845 | 0.485 | 0.365 | 0.335 | 0.385 | 0.850 | 0.380 | 0.290 | 0.515 | 0.285 | 0.200 | 0.455 | 0.285 | 0.270 | 0.795 | 0.575 | 0.535 | 0.535 |
| Phylum | Chloroflexi |  |  |  |  |  |  |  |  |  |  |  |  |  |  |  |  |  |  |  |  |  |  |
| Family | Anaerolineaceae |  |  |  |  |  |  |  |  |  |  |  |  |  |  |  |  |  |  |  |  |  |  |
| Genus | uncultured | 0.410 | 0.325 | 0.310 | 0.250 | 0.400 | 0.225 | 0.605 | 0.305 | 0.270 | 0.310 | 0.420 | 0.215 | 0.260 | 0.140 | 0.475 | 0.465 | 0.540 | 0.190 | 0.175 | 0.215 | 0.000 | 0.000 |
| Phylum | Fibrobacteres |  |  |  |  |  |  |  |  |  |  |  |  |  |  |  |  |  |  |  |  |  |  |
| Genus | Fibrobacteres | 1.590 | 1.245 | 3.450 | 2.475 | 1.925 | 1.125 | 2.775 | 1.025 | 6.595 | 6.670 | 2.080 | 1.595 | 3.305 | 2.155 | 3.340 | 1.380 | 5.625 | 1.200 | 8.030 | 3.640 | 9.670 | 9.670 |
| Phylum | Firmicutes |  |  |  |  |  |  |  |  |  |  |  |  |  |  |  |  |  |  |  |  |  |  |
| Family | Streptococcaceae |  |  |  |  |  |  |  |  |  |  |  |  |  |  |  |  |  |  |  |  |  |  |
| Genus | Streptococcus | 0.000 | 0.000 | 0.000 | 0.000 | 0.000 | 0.000 | 0.115 | 0.205 | 0.255 | 0.170 | 0.155 | 0.180 | 0.215 | 0.575 | 0.225 | 0.260 | 0.180 | 0.405 | 0.125 | 0.000 | 0.000 | 0.000 |
| Family | Family_XIIIIncertaeSedis |  |  |  |  |  |  |  |  |  |  |  |  |  |  |  |  |  |  |  |  |  |  |
| Genus | Incertae_Sedis | 0.720 | 0.350 | 0.625 | 0.365 | 0.485 | 0.215 | 0.290 | 0.210 | 0.275 | 0.255 | 0.160 | 0.155 | 0.210 | 0.000 | 0.545 | 0.260 | 0.300 | 0.135 | 0.000 | 0.000 | 0.000 | 0.000 |
| Genus | Mogibacterium | 1.895 | 1.250 | 1.980 | 0.865 | 2.375 | 1.390 | 1.540 | 1.035 | 1.235 | 1.020 | 1.215 | 0.855 | 1.225 | 0.915 | 2.155 | 1.080 | 1.560 | 0.805 | 0.470 | 0.255 | 0.585 | 0.585 |
| Genus | uncultured | 1.340 | 0.790 | 1.305 | 0.615 | 1.565 | 0.850 | 1.170 | 0.700 | 1.370 | 1.210 | 0.760 | 0.445 | 0.660 | 0.410 | 1.735 | 0.815 | 1.520 | 0.395 | 0.410 | 0.260 | 0.600 | 0.600 |
| Family | Lachnospiraceae |  |  |  |  |  |  |  |  |  |  |  |  |  |  |  |  |  |  |  |  |  |  |
| Genus | 013F01_B_SD_P15 | 0.500 | 0.325 | 0.430 | 0.250 | 0.230 | 0.235 | 0.170 | 0.170 | 0.210 | 0.260 | 0.290 | 0.170 | 0.415 | 0.580 | 0.190 | 0.180 | 0.235 | 0.220 | 0.245 | 0.000 | 0.000 | 0.000 |
| Genus | Acetitomaculum | 6.300 | 3.565 | 6.410 | 2.325 | 5.160 | 2.495 | 2.925 | 1.700 | 2.800 | 2.480 | 5.615 | 1.170 | 1.360 | 1.065 | 2.525 | 1.385 | 2.220 | 1.775 | 0.895 | 0.535 | 1.080 | 1.080 |
| Genus | Acetivibrio_ethanolgignens | 0.160 | 0.155 | 0.215 | 0.220 | 0.210 | 0.195 | 0.415 | 0.245 | 0.540 | 0.520 | 0.380 | 0.305 | 0.440 | 0.195 | 0.385 | 0.350 | 0.390 | 0.195 | 0.205 | 0.105 | 0.000 | 0.000 |
| Genus | Blautia | 0.405 | 0.385 | 0.545 | 0.385 | 0.890 | 0.610 | 0.905 | 0.250 | 1.585 | 1.245 | 0.695 | 1.415 | 0.755 | 0.845 | 1.095 | 1.165 | 1.340 | 0.445 | 0.550 | 0.330 | 0.705 | 0.705 |
| Genus | Butyrivibrio | 2.505 | 1.335 | 2.480 | 1.540 | 2.165 | 1.295 | 2.005 | 2.075 | 2.740 | 2.605 | 4.305 | 1.445 | 4.135 | 2.345 | 1.655 | 1.020 | 1.560 | 1.965 | 2.140 | 0.805 | 2.775 | 2.775 |
| Genus | Butyrivibrio_fibrisolvens_H15 | 7.960 | 4.785 | 7.520 | 3.505 | 8.135 | 10.955 | 7.755 | 5.845 | 6.525 | 5.795 | 8.260 | 7.120 | 7.370 | 7.395 | 8.240 | 6.535 | 8.225 | 6.460 | 2.650 | 2.355 | 4.725 | 4.725 |
| Genus | IS_C_phytofermentans | 0.380 | 0.245 | 0.620 | 0.410 | 0.390 | 0.125 | 0.395 | 0.205 | 0.685 | 0.525 | 0.205 | 0.440 | 0.835 | 0.545 | 0.720 | 0.425 | 0.700 | 0.250 | 0.400 | 0.340 | 0.500 | 0.500 |
| Genus | IS_Eub_cellulosolvens | 0.000 | 0.000 | 0.000 | 0.000 | 0.000 | 0.000 | 0.110 | 0.000 | 0.000 | 0.145 | 0.515 | 0.120 | 0.215 | 0.000 | 0.000 | 0.000 | 0.120 | 0.155 | 0.110 | 0.000 | 0.000 | 0.000 |
| Genus | IS_Eub_hallii | 0.500 | 0.330 | 0.505 | 0.215 | 0.640 | 0.340 | 0.415 | 0.195 | 0.345 | 0.250 | 0.160 | 0.440 | 0.160 | 0.240 | 0.290 | 0.525 | 0.335 | 0.285 | 0.100 | 0.190 | 0.340 | 0.340 |
| Genus | IS_Eub_rum_Coprococcus_A2_166 | 0.200 | 0.230 | 0.440 | 0.260 | 0.175 | 0.000 | 0.210 | 0.225 | 0.545 | 0.500 | 0.525 | 0.270 | 0.735 | 0.510 | 0.330 | 0.360 | 0.315 | 0.350 | 0.190 | 0.215 | 0.900 | 0.900 |
| Geneus | IS_cTPY-17_adhufec52 | 0.405 | 0.245 | 0.550 | 0.405 | 0.445 | 0.270 | 0.385 | 0.325 | 0.540 | 0.435 | 0.410 | 1.290 | 0.490 | 0.825 | 0.395 | 0.735 | 0.475 | 0.525 | 0.325 | 0.620 | 0.655 | 0.655 |
| Genus | Marvinbryantia | 0.600 | 0.350 | 0.520 | 0.205 | 0.685 | 0.350 | 0.790 | 0.265 | 0.945 | 0.570 | 0.465 | 0.425 | 0.420 | 0.465 | 0.870 | 0.445 | 0.635 | 0.225 | 0.295 | 0.200 | 0.730 | 0.730 |
| Genus | Moryella | 0.495 | 0.375 | 0.380 | 0.250 | 0.835 | 0.630 | 0.320 | 0.200 | 0.255 | 0.235 | 0.000 | 0.285 | 0.125 | 0.265 | 0.315 | 0.250 | 0.170 | 0.000 | 0.000 | 0.145 | 0.000 | 0.000 |
| Genus | Oribacterium | 0.265 | 0.240 | 0.280 | 0.300 | 0.270 | 0.180 | 0.180 | 0.355 | 0.385 | 0.370 | 0.515 | 0.395 | 0.350 | 0.495 | 0.270 | 0.250 | 0.290 | 0.900 | 0.190 | 0.000 | 0.000 | 0.000 |
| Genus | Parasporobacterium | 0.585 | 0.620 | 0.400 | 0.635 | 0.640 | 0.670 | 0.850 | 0.615 | 0.855 | 1.450 | 1.175 | 0.620 | 1.300 | 0.475 | 0.410 | 0.560 | 0.440 | 0.510 | 0.360 | 0.345 | 0.580 | 0.580 |
| Genus | Pseudobutyrivibrio | 1.090 | 0.740 | 1.135 | 0.690 | 0.705 | 0.465 | 0.145 | 0.375 | 0.250 | 0.285 | 0.375 | 0.330 | 0.725 | 0.550 | 0.250 | 0.245 | 0.325 | 0.270 | 0.390 | 0.150 | 0.000 | 0.000 |
| Genus | RC25 | 0.300 | 0.165 | 0.390 | 0.260 | 0.240 | 0.215 | 0.470 | 0.350 | 0.440 | 0.430 | 0.375 | 0.405 | 0.490 | 0.390 | 0.390 | 0.270 | 0.375 | 0.355 | 0.515 | 0.270 | 0.500 | 0.500 |
| Genus | RC39 | 0.170 | 0.155 | 0.225 | 0.210 | 0.215 | 0.160 | 0.485 | 0.245 | 0.520 | 0.685 | 0.810 | 0.425 | 0.620 | 0.405 | 0.360 | 0.345 | 0.315 | 0.375 | 0.355 | 0.245 | 0.665 | 0.665 |
| Genus | RF38 | 0.210 | 0.205 | 0.280 | 0.180 | 0.255 | 0.130 | 0.400 | 0.280 | 0.580 | 0.380 | 0.560 | 0.315 | 0.415 | 0.350 | 0.375 | 0.240 | 0.625 | 0.215 | 0.665 | 0.810 | 2.940 | 2.940 |
| Genus | RFN8-YE57 | 10.600 | 8.195 | 9.020 | 6.775 | 10.495 | 7.385 | 11.585 | 8.630 | 12.270 | 11.761 | 11.930 | 8.040 | 11.000 | 11.155 | 15.080 | 12.455 | 13.450 | 11.750 | 7.790 | 4.095 | 9.735 | 9.735 |
| Genus | Roseburia | 0.215 | 0.225 | 0.300 | 0.265 | 0.305 | 0.250 | 0.405 | 0.205 | 0.505 | 0.395 | 0.445 | 0.480 | 0.320 | 0.390 | 0.285 | 0.400 | 0.310 | 0.345 | 0.340 | 0.210 | 0.715 | 0.715 |
| Genus | Sporobacterium | 0.415 | 0.560 | 0.435 | 0.520 | 0.500 | 0.495 | 0.895 | 0.700 | 0.735 | 1.635 | 0.995 | 0.990 | 1.320 | 0.710 | 0.620 | 0.785 | 0.545 | 0.515 | 0.400 | 0.420 | 0.000 | 0.000 |
| Genus | Syntrophococcus | 0.200 | 0.160 | 0.280 | 0.000 | 0.300 | 0.225 | 0.500 | 0.145 | 0.610 | 0.380 | 0.375 | 0.345 | 0.325 | 0.305 | 0.545 | 0.345 | 0.525 | 0.190 | 0.310 | 0.150 | 0.000 | 0.000 |
| Genus | uncultured | 0.370 | 0.260 | 0.525 | 0.390 | 0.475 | 0.280 | 0.780 | 0.465 | 0.760 | 0.790 | 0.675 | 0.675 | 0.905 | 0.685 | 0.600 | 0.480 | 0.580 | 0.460 | 0.530 | 0.320 | 1.045 | 1.045 |
| Genus | wet75 | 0.780 | 0.555 | 0.905 | 0.545 | 1.050 | 0.725 | 1.055 | 0.685 | 1.220 | 1.395 | 1.000 | 1.085 | 1.055 | 0.710 | 0.825 | 1.000 | 0.990 | 0.590 | 0.580 | 0.310 | 1.150 | 1.150 |
