# Supplementary material for: Comparative analysis of the metabolically active microbial communities in the rumen of dromedary camels under different feeding systems using total rRNA sequencing
Source: PeerJ. 2020 Oct 29;8:e10184. doi: 10.7717/peerj.10184 (PMC7603790; doi:10.7717/peerj.10184)
Supplement: Supplemental Information 4 [file peerj-08-10184-s004.docx]

**Supplementary Table S4:** Pairwise comparison of the abundance of active bacterial, archaeal, Protozoal, and fungal genera in the rumen of camels distributed on three groups based on the feeding system.

|  | Permutation No. | P-value | PERMANOVA-Pairwise comparison | | | | | | | |
| --- | --- | --- | --- | --- | --- | --- | --- | --- | --- | --- |
|  |  |  | Uncoreccted P-value | | | | Bonferroni-corrected P-value | | | |
| Bacteria | 9999 | 0.0025 | Group | G1 | G2 | G3 | Group | G1 | G2 | G3 |
|  |  |  | G1 |  | 0.0206 | 0.1011 | G1 |  | 0.0618 | 0.3033 |
|  |  |  | G2 | 0.0206 |  | 0.0048 | G2 | 0.0618 |  | 0.0144 |
|  |  |  | G3 | 0.1011 | 0.0048 |  | G3 | 0.3033 | 0.0144 |  |
|  |  |  |  |  |  |  |  |  |  |  |
|  |  |  | Group | G1 | G2 | G3 | Group | G1 | G2 | G3 |
| Archaea | 9999 | 0.0018 | G1 |  | 0.3036 | 0.0247 | G1 |  | 0.9108 | 0.0741 |
|  |  |  | G2 | 0.3036 |  | 0.0011 | G2 | 0.9108 |  | 0.0033 |
|  |  |  | G3 | 0.0247 | 0.0011 |  | G3 | 0.0741 | 0.0033 |  |
|  |  |  |  |  |  |  |  |  |  |  |
|  |  |  | Group | G1 | G2 | G3 | Group | G1 | G2 | G3 |
| Protozoa | 9999 | 0.0001 | G1 |  | 0.0006 | 0.0058 | G1 |  | 0.0018 | 0.0174 |
|  |  |  | G2 | 0.0006 |  | 0.0004 | G2 | 0.0018 |  | 0.0012 |
|  |  |  | G3 | 0.0058 | 0.0004 |  | G3 | 0.0174 | 0.0012 |  |
|  |  |  |  |  |  |  |  |  |  |  |
|  |  |  | Group | G1 | G2 | G3 | Group | G1 | G2 | G3 |
| Fungi | 9999 | 0.0067 | G1 |  | 0.0054 | 0.0574 | G1 |  | 0.0162 | 0.1722 |
|  |  |  | G2 | 0.0054 |  | 0.034 | G2 | 0.0162 |  | 0.102 |
|  |  |  | G3 | 0.0574 | 0.034 |  | G3 | 0.1722 | 0.102 |  |
