# Supplementary material for: Comparative analysis of the metabolically active microbial communities in the rumen of dromedary camels under different feeding systems using total rRNA sequencing
Source: PeerJ. 2020 Oct 29;8:e10184. doi: 10.7717/peerj.10184 (PMC7603790; doi:10.7717/peerj.10184)
Supplement: Supplemental Information 5 [file peerj-08-10184-s005.docx]

Supplementary Figure 1: Comparison of overall relative abundance of bacteria, archaea, protozoa, and fungi in the rumen solid (SF) and liquid (LF) fractions in four camel groups (G1, G2, G3).
